# Adolescent siblings of children with cancer: a qualitative study from a salutogenic health promotion perspective

**DOI:** 10.1080/17482631.2020.1842015

**Published:** 2020-11-08

**Authors:** Birgit Løkkeberg, Ragnhild Sollesnes, Jorun Hestvik, Eva Langeland

**Affiliations:** a Department of Health and Caring Science, Faculty of Health and Social Sciences, Western Norway University of Applied Sciences, Bergen, Norway; b Children’s Clinic, Department of Cancer and Haemathology, St. Olavs Hospital, Trondheim University Hospital, Trondheim, Norway

**Keywords:** Childhood cancer, health promotion, salutogenesis, sense of coherence, siblings

## Abstract

**Purpose:**

To explore the experiences of adolescents with a sibling suffering from cancer from a salutogenic health promotion perspective.

**Methods:**

Seven female siblings aged 13–17 years were interviewed. The interviews were transcribed and subjected to qualitative content analysis.

**Results:**

The analysis findings adhered largely to one main theme: *new challenges and needs in everyday life*, consisting of the two domains *cancer into life* and *helpful resources to cope*. Categories related to the cancer into life domain were *new routines and concerns, fear, loneliness*, and *growing up*. Helpful resources to cope were categorized as *support from others, understanding, faith and hope*, and *normal activities.*

**Conclusion:**

Prominent in the siblings’ descriptions were the challenging changes in everyday life including difficult feelings such as loneliness, and a need for understanding. Social support appeared as a crucial salutogenic coping resource to achieve understanding, faith and hope, and identity—crucial spheres to achieve meaning in life. This study has demonstrated the significance of salutogenesis in a new setting, and the findings could be of relevance to teachers and health professionals consulting with the siblings of children with cancer. Further research should be conducted to pinpoint concrete health-promoting measures that might benefit this group.

## Introduction

Each year, 215,000 children and adolescents aged 0–18 years are diagnosed with cancers worldwide. In Europe, 80% of these children survive (World Health Organization &; International Agency for Research on Cancer, 2016), after a comprehensive treatment with many side effects. Thus, many children and adolescents live with a sister or a brother who is undergoing or has finished cancer treatment.

Focus on siblings of children with severe illnesses is increasing and, in Norway, The Health Personnel Act (1999) §10a was expanded—valid from 2018—to ensure that siblings of children with severe illnesses receive proper information and attention of their own. Siblings’ needs are also mentioned in the Norwegian Government’s strategy for adolescents’ health (Ministry of Health and Care Services, 2016), and in national guidelines dealing with palliative care for children and adolescents (The Norwegian Directorate of Health, 2016), and caring for next-of-kin (The Norwegian Directorate of Health, 2015). The national guidelines for school health services strongly recommend that school nurses are made aware of exposed children and adolescents, and severe illness in the family is mentioned as a life event that might cause mental problems (The Norwegian Directorate of Health, 2018).

When a child or an adolescent gets cancer, all members of the family are affected (Neville et al., 2016; Prchal & Landolt, 2012; Yang et al., 2016), and the normal, secure family life can break apart to be replaced by an everyday life without foothold, safety, or control (Björk et al., 2005). Common emotional responses among siblings are shock, fear, uncertainty, sadness, helplessness, anger, jealousy, and guilt (D’Urso et al., 2017; Long et al., 2018). Siblings can experience sorrow after being told about the diagnosis, sadness for their sick sibling’s loss of a normal life, and sadness about feeling unimportant and forgotten in the family (Nolbris et al., 2013). Further, siblings have reported changes at school, in extracurricular activities, and with friends (Samson et al., 2016). Reduced cognitive and emotional quality of life (QoL) for siblings 1–2 months after the diagnosis have been described (Houtzager et al., 2005), in addition to transiently increased levels of self-reported anxiety 3 months after diagnosis (Lähteenmäki et al., 2004). On the other hand, the changes in everyday life can have a positive effect on siblings, making them more responsible, independent, mature, and empathic (D’Urso et al., 2017; Neville et al., 2016; Yang et al., 2016).

The siblings’ emotional responses and experiences can be influenced by internal and external factors. Increased levels of self-control and self-efficacy are related to lower levels of anxiety and fewer psychosomatic symptoms (Hamama et al., 2008), and the siblings’ age and the time elapsed after the diagnosis might influence their use of coping strategies (Turner-Sack et al., 2016). Good family and peer relationships seem to be important coping strategies (D’Urso et al., 2017; Prchal & Landolt, 2012; Toft et al., 2019). Studies have found a correlation between family function and relationships with their parents, and the development of difficulties with the siblings (Long et al., 2013; McDonald et al., 2015), as well as the siblings’ health-related QoL (Kobayashi et al., 2015). Unmet needs for social support and friendships are associated with depression, behaviour and attention problems, lower QoL, and worse school functioning (Long et al., 2018).

Studies have investigated the long-term effect on siblings’ mental health and QoL. Thus, siblings of long-term survivors generally have good mental health, although some groups are at increased risk of long-term psychological distress (Buchbinder et al., 2011). One study found that 75% of siblings had one symptom or more of post-traumatic stress symptoms (PTS) affecting their function; 22% met the criteria for posttraumatic stress disorder, and, in addition, there was a comorbidity between PTS, and anxiety and depression (Kaplan et al., 2013). Siblings of the survivors of childhood leukaemia have reported increased psychological QoL and decreased social QoL, and higher ages for both patients and siblings might increase the risk of impaired psychological QoL (Berbis et al., 2015). Increased school absenteeism after completion of treatment has been described for survivors as well as siblings (French et al., 2013). The families have been described as still vulnerable and in need of support for up to 7 years after the diagnosis (Sundler et al., 2013). Adult siblings of childhood cancer survivors have reported an increased incidence of risky alcohol consumption, associated with symptoms of depression, anxiety, and psychiatric distress (Lown et al., 2013).

Caring for children with cancer comprises the whole family (Zegaczewski et al., 2016). The siblings’ adaptation to the disease is described as an active process (Long et al., 2015), and contributing factors are suggested. Several studies have pinpointed the need for interventions for siblings (D’Urso et al., 2017; Franklin et al., 2018; Kobayashi et al., 2015; Long et al., 2015; Lown et al., 2013; McDonald et al., 2015; Toft et al., 2019). Early support to siblings seems important in preventing later mental difficulties (Lähteenmäki et al., 2004), and siblings need information, communication, and involvement (Lövgren et al., 2016; O’Shea et al., 2012). Tasker and Stonebridge (2016) identified eight needs of adolescent siblings of children with cancer: attention and acknowledgement; honest and open family communication; inclusion in the family during treatment; knowing that difficult emotions and thoughts are normal; specific emotional support; instrumental support; being children; and sharing family humour, laughter, and light-heartedness. They suggested that specific support should be available to these siblings. Patterson et al. (2017) found a strong positive correlation between levels of distress and the number of siblings’ unmet needs. The frequently reported unmet needs were information, recreation, dealing with feelings, relationship with the sibling with cancer, and support from friends.

Being involved in hospital care can help maintain the sibling relationship and prevent the healthy siblings from feeling excluded (Neville et al., 2016; Toft et al., 2019). Even though health care professionals caring for children with cancer are aware of siblings’ need for information and support, the existing care models, a lack of resources, and siblings’ absence from the hospitals, can make it difficult to offer siblings proper support in the hospital (Franklin et al., 2018). Thus, these siblings might need attention and support from professionals outside the hospital to maintain or promote health. A recently published review article suggested that future research on the siblings of children with cancer should focus on positive outcomes such as health promotion (Long et al., 2018).

Research has improved our knowledge of various difficulties with siblings, both in the short and long term. Personal characteristics, family functioning, social support, and targeted interventions might affect siblings’ mental health. When using salutogenesis as a framework when consulting with affected siblings, knowledge about suitable resistance resources can be helpful in promoting health and preventing difficulties. There is a lack of knowledge about the siblings’ own views on their experiences with resistance resources, which can help them to cope. The aim of this paper was to explore adolescents’ experiences of having a sibling suffering from cancer from a salutogenic health promotion perspective.

## Salutogenesis as a theoretical framework

*Salutogenesis*, defined as “the origins of health” (Antonovsky, 1979, pp. preface, vii), is a robust theory about health promotion (Antonovsky, 1996; Lindström & Eriksson, 2010), and is a beneficial framework for health promotion professionals’ practice. The salutogenesis concept regards a stressor as potentially health promoting when handled in a good way, and the ability to solve tension caused by a stressor to be connected to the person’s *sense of coherence* (SOC). SOC consists of the three inextricably intertwined components: *comprehensibility, manageability*, and *meaningfulness* (Antonovsky, 1979). According to Antonovsky (1987), meaningfulness, which is closely connected to motivation, seems to be the most important component. To maintain or increase meaning in life, the theory emphasizes investing in the following crucial spheres: *inner feelings, social relationships, main activities*, and *existential issues* (Antonovsky, 1987).

Another concept in the theory is *generalized resistance resources* (GRRs), defined as “any characteristic of the person, the group, or the environment that can facilitate effective tension management” (Antonovsky, 1979, p. 99). SOC and GRRs function in mutual interplay. The higher the SOC, the stronger the ability to use GRRs, and when using GRRs, SOC is promoted (Antonovsky, 1987). In addition, the theory also emphasizes the presence of *specific resistance resources* (SRRs) that are used to adapt to particular stressors, and that a stronger SOC increases a person’s ability to choose the best available SRR when meeting a particular stressor (Antonovsky, 1987; Mittelmark et al., 2017).

## Methods

### Study design

A qualitative approach with an explorative design was chosen. To obtain the adolescents’ experiences, individual interviews were performed. The transcribed interview texts were subjected to qualitative content analysis as described by Graneheim and Lundman (2004). This analysis method facilitates understanding the text in different abstraction and interpretation levels. The manifest part of the analysis describes the content in the texts, as it appears in the informants’ own words and phrases. In the latent part of the analysis, the researchers use abstraction and interpretation to find the underlying meaning in the texts, presented in a main theme. Thus, the qualitative content analysis describes and analyses the content as it is experienced and expressed by the informants and interpreted by the researchers.

The design chosen for this study corresponds to a phenomenological and hermeneutic point of view, as discussed by Graneheim et al. (2017).

### Sample

Siblings eligible for inclusion were aged 12–20 years, and having a sister or a brother who was undergoing or had finished and survived treatment for childhood cancer, diagnosed at age 0–16 years. Adolescents were chosen as informants in the present study because from 12 years of age, adolescents have the right to be heard on health issues (The Health Personnel Act, 1999) and they have the ability to more complex and abstract thinking than younger children (Piaget et al., 1974).The term “sibling” in this study (cf. the inclusion criteria) comprises biological, half-, step-, and adopted siblings. Siblings could participate if they lived together with their sick sister or brother, full or part time, during the illness period, and understood and spoke the Norwegian language properly. Exclusion criteria were severe illnesses among the siblings themselves or others in the family, and impaired cognitive function.

All eligible siblings of childhood cancer patients treated in one of five Norwegian university hospitals during the last 5 years were asked to participate. A nurse in the hospital department made a telephone call to parents and received their permission to send written information with a consent form to their daughter or son. In some cases, the forms were delivered to parents in the hospital, who brought them along to their children. Siblings who agreed to participate returned the consent directly to the first author in an attached franked envelope. Participants aged 16 years or older gave their own consent, while the younger ones signed together with their parents. Parents in 16 families gave their permission to ask their sons or daughters to participate, but siblings in only three families agreed. Therefore, the Norwegian Children’s Cancer Foundation was contacted and asked to contribute to the recruitment. The foundation is run by parents of cancer-suffering children, and they asked eligible siblings for participation. Siblings willing to participate contacted the first author, who sent them written information with the consent form. Four siblings were recruited through the foundation. All seven informants were girls; they were all older than their siblings with cancer; their age at the interview time was 13–17 years. Four informants were biological siblings of the children with cancer, three were half-siblings, and they all lived with their siblings with cancer most of the time. Time elapsed after the diagnosis was 1–5 years. The informants’ siblings with cancer were all boys, their age at the interview time was 2–14 years, and their cancer treatments had been completed. The informants lived in four different counties in two of five Norwegian health regions, and the interviewer visited their home places to perform the interviews.

### Data collection

Data were collected through seven individual interviews performed by the first author from September to November 2017. The interviews lasted 21–40 minutes for a mean of 31 minutes. To ensure integrity and autonomy, time and place were chosen by the participants. Five interviews took place in informants’ homes, one in a public office, and one in a library. A semistructured interview guide covering the themes and questions we desired to have answers to was used. To explore these informants’ experiences from a salutogenic health promoting perspective, the informants were asked to describe their experiences, and more importantly, to describe what had made the experiences easier. The two open-ended questions: “Can you describe what it felt like to learn that your brother has cancer?” and “Has anything specific helped you to cope?” initiated the two overall themes, and were asked in all the interviews. Further, the informants told about their experiences in their own words. Except from the two main questions, the interview guide consisted of suggested follow-up questions and served as a checklist with some points. These points represented themes like what they needed when their sibling was sick, how they experienced their relations with friends and family, and what was most important to them. Additional themes that aroused were followed up thoroughly by the interviewer.

Quality criteria for interviews are the use of short questions and spontaneous, rich, specific, and relevant answers, following up the questions, and, interpretation and verification during the interviews (Brinkmann & Kvale, 2015). The interviewer endeavoured to adhere to these criteria, as well as appearing knowledgeable, organized, clear, sensitive, and open, all of which are mentioned as interviewing skills (Brinkmann & Kvale, 2015). Before the interviews ended, the content was summarized by the interviewer to ensure that she had perceived everything correctly, and the informants were asked if there was anything else they would like to say.

All the interviews were audio recorded and shortly afterwards—within 24 hours—they were transcribed verbatim by the interviewer.

### Data analysis

Data were analysed using qualitative content analysis (Graneheim & Lundman, 2004), and the entire transcribed texts constituted the analysis unit. Initially, the first, second, and last authors read the entire texts to become familiar with the content and achieve an overall impression. Then, the text was sorted into the two domains, or content areas, *cancer into life* and *helpful resources to cope*, based on the two main questions in the interview guide. Meaning units—sentences related to the aim of the study—were identified and condensed. The condensed meaning units were coded further and sorted into subcategories and categories representing the manifest content. These categories led to a main theme, representing the latent content, which was interpreted and discussed by the authors. Table I gives examples of the steps in the analysis, from meaning unit to category. The first, second, and last authors contributed throughout the analysis by discussing the steps in the analysis and working out a consensus.

**Table I.** Examples of the analysis, from meaning unit to category

Meaning unit example quotes	Condensed meaning unit	Code	Subcategory	Category
“Actually, I felt quite alone. I felt I had no one to talk to. So, I felt quite alone on this earth. That I was the only person who understood myself. And no one understood me. And I thought it was very difficult.”	Felt lonely, with no one to talk to and no one who could understand	No one to talk to; no one could understand	No one understands	Loneliness
“I felt like I wasn’t on the same human level as them.”	Being on another level from the others	On a different level	Feeling different	
“I felt everything was inside, that is, everything was held in. I couldn’t let it out.”	Keeping thoughts inside unable to let them out	Keeping thoughts inside	Alone with thoughts	
“I pushed my friends away, so that I wouldn’t bother them or make them sad.”	Pushing friends away to avoid bothering them and making them sad	Pushing friends away to not bother them	Pushing friends away	
“All the focus is on the sick one, but actually someone else is standing beside them and needs some attention, too.”	Standing beside, needing attention	Feeling forgotten	Jealousy	

### Methodological considerations

Lincoln and Guba (1985) have suggested four criteria for enhancing trustworthiness in qualitative research: *credibility, transferability, dependability*, and *confirmability*.

To achieve credibility, it is important to find participants who have experience and can tell about the phenomenon being studied (Graneheim et al., 2017). To explore and describe siblings’ experiences, the siblings themselves were recruited, and open questions were chosen as an appropriate approach to obtain the most unpredictable data. The analysis process has been described, and the presentation of the findings is supplemented by quotes.

To facilitate transferability, it is important to have good descriptions of context, selection, data collection, and analysis. In addition, the findings should be presented in a thorough and substantiated manner. Nevertheless, the readers must decide whether the findings are transferable (Graneheim & Lundman, 2004). This study has endeavoured to meet the above criteria so that transferability can be assessed.

Dependability is a prerequisite for credibility and refers to the stability of data, meaning that another researcher would achieve the same findings with the same participants (Polit & Beck, 2017). The researcher’s preunderstanding might influence the findings through how the questions are asked and followed up, and how the interviews are interpreted (Graneheim et al., 2017). The interviews were conducted by the first author, who had experience as a nurse in a childhood cancer hospital department. Thus, preunderstanding can be seen as one of the motivational factors for the study. The interviewer’s proximity to the topic was helpful to achieve contact and confidence in the interview situation, and to understand the context. At the same time, the interviewer had the necessary objective distance from the field because several years had elapsed since she had been working in a hospital setting. The interviewer listened carefully, followed up on topics that appeared, and summed up to ensure she had perceived correctly. To achieve trustworthy data, the first, second, and last authors read the interviews and participated throughout the analysis.

Confirmability refers to the objectivity and possibility of congruence between several independent people about the accuracy, relevance, or significance of the data, and whether the data accurately reflect what the informants said in the interviews (Polit & Beck, 2017). To achieve confirmable findings in this study, preunderstanding was reflected upon and described. The selection of informants, data collection and analysis were also described.

### Ethical considerations

The study was designed and performed following the principles of the Declaration of Helsinki (World Medical Association, 2013), and was approved by the Western Norway Regional Medical and Health Ethics Committee (REC West, 2017/1180). Voluntary participation and the permission to withdraw at any time without any consequences were clearly emphasized, both in the written and oral information. Before each interview started, the participant was informed about the possibility of pausing or interrupting it. It was suggested that school nurses at the informants’ schools could be informed about participation in the study so that they could be consulted. None of the informants requested this.

## Findings

### New challenges and needs in everyday life

The siblings’ experiences were described in the two domains *cancer into life* and *helpful resources to cope*. Each of these domains consisted of four categories with associated subcategories. Table II shows domains, subcategories, categories, and the main theme. The informants described how extensive changes in their daily lives influenced both their feelings and their relationships with others. In addition, they described how they handled these changes in various ways. The categories led to the main theme: *New challenges and needs in everyday life*.

**Table II.** Domains, subcategories, categories, and main theme

Domains	Subcategories	Categories	Main theme
Cancer into life	Separation Changed family life	New routines and concerns	New challenges and needs in everyday life
	Scary and painful time Reactions Hard to concentrate Hard to sleep Strong impressions Thinking of death Reminders	Fear	
	No one understands Feeling different Alone with thoughts Pushing friends away Jealousy	Loneliness	
	Increased responsibility Becoming stronger Increased empathy Expanded life perspectives	Growing up	
Helpful resources to cope	Friends Classmates Being seen Other siblings Stable and caring persons	Support from others	
	Information Time to understand	Understanding	
	Knowing everything was good Thinking positively Having faith in treatment Praying to God	Faith and hope	
	Being at school Being with family	Normal activities	

### Cancer into life

The domain *cancer into life* consisted of four categories; *new routines and concerns, fear, loneliness*, and *growing up.*

#### New routines and concerns

The siblings described how their lives were turned upside down suddenly when they learned that their brothers had developed cancer. Cancer entering their lives affected the siblings in several ways and brought new concerns. One informant said that she did not know anything about cancer a couple of years ago. Now, she saw cancer “everywhere” and was aware of it every day.

The siblings were separated from their parents and families for varying durations, while others in the family stayed in the hospital:

… and then they lived their lives at the hospital, and we lived our lives at home (7).

Some siblings stayed at home with grandparents and other healthy siblings, some stayed with friends, and some stayed “everywhere.” Everyday life became different and unfamiliar:

It was … quite different. Normally we go to school, mom and dad go to work, and later we all go home, and so on (7).

Siblings’ responses to the separation varied. While some felt left alone and lonely to a great extent and missed their families very much, others managed better.

The siblings described several changes in their families as a result of the cancer diagnosis. The main focus in the families was moved from the usual issues to illness and treatment. The siblings could be affected both by the condition of the child with cancer and others in their family. One informant expressed her worries about her mother, who had been very tired, and she described that everything became easier when she noticed that her mother was feeling better. Another expressed her concern about her brother:

I could sleep, but I thought of him when I woke up (2).

Even while others in the family were at home occasionally, siblings had to be more careful than before, and they could not act as they usually did with the family. They told about precautions because of the risk of infections, having to be quiet and careful all the time, and being unable to be themselves completely, and these things could make them tired of the situation.

Mom and dad were irritated with us because we didn’t take enough care, although we thought we were quite careful. Everyone was a bit tired (7).

#### Fear

The time around the diagnosis was described as scary and painful. The diagnosis was a shock, everything felt new, strange, and hard to believe, and some siblings did not know how to react. Reactions within the family could be different:

I started to cry, while my sister didn’t cry … then I saw Dad cry. I had never seen Dad crying before. So, it was really surreal. It really was a shock. I was terrified (6).

Informants described themselves as vulnerable, anxious, and sad, and they felt afraid, worried, and curious about what would happen. It could be hard to concentrate at school and to sleep at night. Others did not manage to understand the seriousness of the situation or respond at all. The cancer itself could be both painful and scary:

… and I didn’t dare to go in to him, so I stood outside. It lasted about 20 minutes until I dared to enter his room (5).

The siblings also had to deal with cancer as a potentially fatal disease:

In the beginning, I thought that he could die, but I didn’t tell anyone. Although the prognosis was good, I knew there was a possibility that he could die (3).

It hurt to think that he could actually die … that we did not know what could happen (4).

Siblings were aware of the nature of the cancer and afraid of relapse for a long time after the treatment was finished. Reminders, expressed as strong images, could appear several years afterwards:

Sometimes I can have something like flashbacks, I relive everything again … As if I live there again … Of course, then I start to cry (5).

#### Loneliness

The siblings could feel lonely, mainly because they thought no one could understand:

Actually, I felt quite alone. I felt I had no one to talk to. So, I felt quite alone on this earth. That I was the only person who understood myself. And no one understood me. And I thought it was very difficult (5).

Trying to make others understand was described as stressful, and some did not talk to friends because they found it quite difficult to explain. When friends expressed that they understood, without actually doing so, it could increase the feeling of loneliness and sadness.

The siblings could feel different from others. One informant felt that she was “being on another human level” (3). Another expressed it like this:

It is almost like there are two different types of people—the ones with cancer in the family—and all the others (4).

It could be difficult for the siblings to share their thoughts and feelings with others. One reason for this was that they thought friends did not understand:

It’s difficult talking to friends, because they hardly know what cancer is (1).

It isn’t easy to talk to friends about how it is. Because they don’t understand (4).

One informant said that she kept everything inside her mind because she was afraid to say something wrong and make things worse:

I felt everything was inside, that is, everything was held in. I couldn’t let it out. I was really struggling (5).

One sibling described a situation at school when she started to cry and was left by her teacher to comfort herself, sitting alone with many unanswered questions.

Some siblings would not “bother” friends by talking about their feelings because they might not know what to say, and they were worried about pushing their friends away. One informant was worried about making others sad:

I pushed the friends away, because I wouldn’t bother them or make them sad (3).

Siblings could feel jealous and lonely within the family, as a result of the sick children’s needs for attention and care:

You are very forgotten. Because all the focus is on the one who is ill, while there actually is someone outside who needs some care, too (1).

I felt that my little sister was forgotten sometimes, which made me sad. Of course, the parents have to focus on the sick child, but at the same time it is important not to forget their other children (3).

#### Growing up

Siblings were given increased responsibility to plan and organize their changed daily lives, in their parents’ absence:

I had to grow up very fast, I was 12 years old, and I had to manage myself. I had to pack my clothes and toiletries and such things myself. And then I had to make sure somebody could wash those clothes, so I had to ask about it … had to ask to be driven here and there, to football training, and so on. It was not a matter of course anymore, I had to ask around (1).

Siblings also described growing and developing themselves, becoming stronger and more resistant than before, and gaining increased ability to understand others:

I think I have learned to feel it when people are suffering. When my brother was sick, no one could see that I was suffering, although I did not feel well inside. This made me aware that people might be sad inside, even though they seem happy outside (7).

In addition, siblings used words describing new and expanded life perspectives for appreciating themselves and others as being healthy.

Earlier, I didn’t think about whether people were healthy—normally, people don’t think about such things. When my brother was ill, I thought that I was glad that people were healthy, and I became more grateful for what I had around me. I don’t think my friends can see this before they experience it themselves. I think no one does (6).

### Helpful resources to cope

The siblings dealt with cancer entering their lives in various ways. This domain consisted of the categories *support from others, understanding, faith and hope*, and *normal activities.*

#### Support from others

Informants considered that having someone to talk to when they needed it was the most important factor helping them to cope during the illness period.

The most important thing to me was having the opportunity to talk to someone if I needed it (2).

In addition to family members, who were most important, they could get support from others. Staying with friends could be difficult—as mentioned previously—but also good and helpful. Being active with friends could make the siblings think of something else other than the disease:

With friends I could think of anything else, be active and have fun (1).

Support from friends could mean a lot, and it felt good to receive hugs and comfort. Friends could be quite honest and say things as they were, unlike adults who could be more protective. The siblings needed to be themselves without anyone feeling sorry for them or having any expectations of how they should cope with the situation:

When I wasn’t that deep down anymore, and people started treating me like they did before, it became much easier for me (3).

It was important not to be identified with the disease, as “she with the sick brother.” Even though friends did not understand exactly how it was, they could understand that it was difficult, and still give support and comfort:

I was very comforted the day after I was told that he was sick. Because, then I told my class at school. Then I didn’t manage to keep my tears inside, so I cried a lot, and they all came together and just … stroked my shoulder, held my hand, and stroked my hair and cheek (4).

Stable and caring persons, such as grandparents, aunts and uncles, their own siblings, or friends of the families, helped to make the experience easier.

Without our grandmother, I don’t see how we could have managed (6).

One sibling shared an experience at the hospital when she had received an iPad purchased by the Childhood Cancer Foundation, delivered by a nurse in the department:

My brother got all the attention, gifts and so on, so actually I was a bit jealous. Then I got a brand new iPad. I became happy, not only because of the iPad itself, but mostly because he (the nurse) saw me (1).

Being with other siblings of children with cancer through the Childhood Cancer Foundation’s family arrangements, could be a good and useful experience:

You get to know once. And there, you don’t feel different. You don’t have to feel it either, because the others, they have experienced the same as you. You can be yourself, and if you have a bad day, they give you space, you don’t need to explain. Because they understand (4).

The most important thing is to know that you are never alone, there are others experiencing exactly the same as you (5).

Meeting others with similar experiences and worries, could create feelings of normality, safety, and comfort.

#### Understanding

The informants reported understanding as a central need and an important prerequisite for having faith and hope. Information given by the hospital staff or the parents could increase their understanding. The siblings’ own understanding could give them confidence to talk to others about what had happened and have the knowledge to answer questions, so this was helpful in relationships with others. Time alone with thoughts could be important to enable understanding:

I needed a little break, some time to understand what happened. It was so much, with everything, school, and friends … Then I relaxed. Just switched off the phone and disconnected for a while. I did not think of school or anything, I just thought about what had happened (6).

Informing the class could increase understanding. While some did not want to share information with their class because they wished everything to stay as normal as possible, others preferred that their classmates be informed about the cancer disease:

I felt a bit strange if I suddenly started crying or left the classroom or went home or spent a day at the hospital … therefore I thought it was okay that my classmates knew why (1).

#### Faith and hope

Knowing that everything was good at the hospital was described as a prerequisite for coping.

One informant reported that, after she had received information about the disease and the treatment at the hospital, she made a conscious decision to think positively, to have faith in the treatment, and to perform her regular activities:

Well, my brother is in the hospital now. I can help him by not being negative, so I will continue with school, hobbies, and such things (2).

During the illness period she was more focused on practical issues than on feelings, and she preferred not to talk to others about the disease.

Praying to God, hoping and believing in a good outcome, and shutting out fears of a bad outcome were examples of positive coping strategies.

I thought that it had to go well, and I never thought that it would not go well, that it was an alternative (7).

#### Normal activities

Some preferred being consecutively informed about what was going on at the hospital. Others benefited from living as normally as possible with distance from the illness and its treatment, and without knowing everything that happened:

I needed to live as usually as possible (7).

Informants also told about how pressure at school could make the days more difficult and make them feel tired. On the other hand, it could be good to be at school:

I thought it was good being at school. Because I got something else to think of (6).

School was the constant, the same as before he got the diagnosis (2).

Good experiences with understanding teachers were described.

If I had to go home because I was worried about my brother, I could just leave. So, they were very understanding (7).

One teacher had made a call to a family member during the summer vacation to prepare for the start of school for the sibling, and to clarify whether she had any extra needs. Others told about teachers being available for talking, and teachers adapting schoolwork and lowering requirements for a while:

“If you need exemption from samples and so, because you have to help at home or something like that, then it’s okay”. Because they understood. That I got tired and had some other things to worry about (7).

Normal family activities were mentioned as important and could make the siblings feel better. Siblings underlined the importance of visiting their families in the hospital, having time with others in the family, and being comforted by their parents. One informant said that she felt everything was very difficult for a while, then she felt better after some days at the hospital with her family:

I stayed there for a few days … with the family … Normal family for a little while (1).

Strong family ties were described and, together with the family, they could cry, smile, laugh, and share feelings, and be completely themselves.

## Discussion

The interviews gave insight into adolescent siblings’ experiences when children develop cancer. The qualitative content analysis led to the main theme: *New challenges and needs in everyday life*. Consciousness on new challenges and needs is important for development of salutogenic coping skills. Responses to the diagnosis were shock and fear, and these emotions could be accompanied by a feeling of loneliness. Siblings were separated from others in the families, and they were given more responsibility than previously. Cancer brought new concerns, both in the short and long term, and personal growth and new perspectives on life were described. Growth and new life perspectives can help to strengthen identity; a crucial GRR in the development of SOC. Relationships within the families, and with friends and teachers were affected, and siblings reported relationships with other siblings of children with cancer as being useful. Social relationships is another GRR that contribute in development of SOC. The informants reported various coping strategies, and some managed better than others. This can be related to the strength of SOC, access to GRRs, and the ability to find and make use of SRRs to solve tensions. Age and stage of development have to be considered as contributing factors.

These findings indicate that childhood cancer represents a comprehensive stressor to the sick children’s siblings. According to the theory of salutogenesis, resolving the tension caused by a stressor might promote coping, meaningfulness, and health. Our findings show that to convert tension into coping, the siblings had to find meaning in regulating their own emotions and feelings, like fear and loneliness, including those on existential issues, and to find meaningful activities. In addition, the quality of social support appeared as a main and superior resistance resource in itself and in promoting coping in all the three abovementioned areas. These findings are compatible with the four spheres: relationships, feelings, activity, and existential issues, which—according to salutogenesis theory—are crucial GRRs to invest in to maintain and promote meaningfulness, the most important component in the SOC.

### Quality of social relationships

An important resistance resource is close emotional relations with other people (Antonovsky, 1979), and previous studies have indicated that social support is a protective factor that may reduce depression and anxiety in this group (Long et al., 2018, 2013; Prchal & Landolt, 2012; Tasker & Stonebridge, 2016). The informants were exposed to a stressor that implied separation from the parents for a period, so that the parents, as resistance resources, became less available to them. At the same time, the siblings became more vulnerable, and their need for support increased. Some received care and support from grandparents, siblings, or others in the family, while others were taken care of outside the family. Previous papers have described the family becoming vulnerable and more dependent on others (Björk et al., 2005), and siblings feeling forgotten within the family (Neville et al., 2016; Nolbris et al., 2013; Toft et al., 2019), which can increase the levels of psychological distress and unmet needs (Patterson et al., 2015).

Siblings given increased responsibility in making arrangements that their parents usually made, corresponds with studies describing changed roles in the family (Neville et al., 2016; Yang et al., 2016), and siblings’ needs to be children and have less responsibility (Tasker & Stonebridge, 2016). The condition of the sick sibling and their parents’ wellbeing influencing the healthy siblings, corresponds with a study that found a strong correlation between the parents’ function and the siblings’ health-related QoL (Kobayashi et al., 2015). Informants reported that time spent with the family and attention from their parents made the situation easier. Family unity represents a coping strategy (D’Urso et al., 2017; Prchal & Landolt, 2012), and there is an association between weakened relationships with parents and a higher incidence of mental disorders and unmet needs with siblings (McDonald et al., 2015).

Relationships with friends could be changed, as also described by Samson et al. (2016). These relationships were described as supportive and useful when the friends treated the siblings as they normally did. Being identified by the disease or being felt sorry for made siblings feel different from others. Siblings appreciating friends acting as usual was also described by Prchal and Landolt (2012), and the need for “time out” and the experience of normality were also reported by Patterson et al. (2017). This also corresponds with the assumption in salutogenesis theory that the whole person’s experience should be emphasized rather than just the problem. This illustrates both the importance of social identity as a GRR, and the connection between social support and identity. Social supports of good quality, tension, active adaptation, and strengthening of identity are factors closely linked with each other (Langeland et al., 2016). When friends expressed their wish to support, this represented an SRR, because the friends adapted to the situation. On the other hand, there were other informants who felt increasingly alone. Being unable to talk to friends, because they could not understand, was stressful. Lacking resistance resources can also be a stressor (Antonovsky, 1979), and others who do not understand can be seen as an autonomous mutual interaction between lack of social support as a resistance resource and a weaker SOC.

The reported good experiences with social support from other siblings of children with cancer are consistent with previous studies that have pointed out the sense of belonging and the importance of sharing experiences in support groups (Neville et al., 2016; Nolbris & Ahlström, 2014). Being together with others in the same situation might be a GRR that strengthens identity, and at the same time an SRR, because it is adapted to the situation.

### Description and regulation of emotions and feelings

The informants gave detailed descriptions of the emotions and feelings they experienced when their siblings were suffering from cancer, indicating that those memories and feelings were still strong, and probably little processed. Reactions were described with words such as *shock, unbelievable*, and *afraid*, as also described by D’Urso et al. (2017) and Long et al. (2018). Dealing with feelings is identified as a central need with siblings (Patterson et al., 2017). Informants reported emotions and feelings associated with the disease as being still present with them after the treatment was completed, in the form of unpleasant pictures in their heads, fear of relapse, and fear of losing their siblings. This finding is consistent with the findings of Kaplan et al. (2013), and Alderfer et al. (2003), both of which found an increased incidence of PTS in adolescent siblings of childhood cancer survivors.

Nurses in the children’s hospital department who provided information, represented an SRR, because they were expedient social resources in this particular situation. The information could give hope that the sick siblings would become well again, and, in addition, the healthy siblings could feel more comfortable with friends because they had some answers to their questions. Thus, information can help facilitate and strengthen social support from friends and prevent loneliness. The importance of being informed corresponds with a study concluding that person-centred interventions with siblings in a hospital department may be helpful for gaining a better understanding, sleeping better, and avoiding bodily ailments (Nolbris & Ahlström, 2014).

The feeling of loneliness was a prominent finding, and has also been described previously (Toft et al., 2019). When siblings had tried to make others understand but in vain, they described these efforts as stressful, and as exacerbating their feeling of loneliness. Lack of social support might cause poor tension management, and probably a weaker SOC (Antonovsky, 1987). The informants were at an age where their SOC was not fully developed, which means that they did not necessarily have access to or ability to use appropriate resistance resources to regulate their feelings. However, some siblings handled their feelings by taking responsibility and organizing their daily activities in a new and adapted manner. This can be related to the assumption of human adaptability by the salutogenesis theory, and is associated with a stronger SOC. The differences in the informants’ descriptions of their feelings can be related both to varying access to and ability to use resistance resources, and to different ages and stages of maturity.

The ability to handle one’s feelings is a prerequisite for being able to mobilize resources to deal with a problem (Antonovsky, 1987). Houtzager et al. (2005) described reduced emotional QoL by siblings the first time after the diagnosis: in other words that their ability to handle feelings might be weakened. Social support is described as important for siblings’ psychological adaption (Long et al., 2018; Neville et al., 2016; Toft et al., 2019), and informants in the present study stated that having someone to talk to was the most helpful resource. Thus, appropriate social support appears as an important SRR helping to regulate feelings.

### Meaningful activities

Having meaningful activities is one of the crucial salutogenic prerequisites for achieving meaning in life (Antonovsky, 1987). Children and adolescents spend much time at school, so this is an important arena. Whether school activities are experienced as meaningful depends largely on the quality of social support from teachers and others at school. The informants reported that concentrating at school and fulfiling the requirements could be difficult, as also described by Alderfer et al. (2015), Prchal and Landolt (2012), and Samson et al. (2016), suggesting that teachers might help in reducing pressure. Some informants reported understanding and supportive teachers, while others described that their needs were neither seen nor understood by their teachers. Generally, teachers can be seen as GRRs, who might promote the pupils’ experience of meaningfulness, comprehensibility, and manageability, and thus contribute to a stronger SOC. When a child or adolescent gets cancer, the teacher might also represent an SRR that is adapted to the particular situation for the siblings. Reduced cognitive QoL for siblings during the first few months after a diagnosis of cancer has been described (Houtzager et al., 2005), in addition to a long-term increased risk of a high rate of school absenteeism (French et al., 2013). In addition, long-term learning problems have been described, and, to avoid these, the school should be informed and facilitate the siblings’ learning (Lähteenmäki et al., 2004). The school, as an environment with meaningful activities, can then represent a resistance resource.

Hobbies and leisure activities with friends helped the siblings think of things other than the disease, as also described by Patterson et al. (2017). Previous studies have shown that siblings are less engaged in leisure activities when children have cancer (Alderfer et al., 2015; Prchal & Landolt, 2012; Samson et al., 2016). This could be a result of the family’s changed situation and parents being less available. Facilitating siblings’ participation in such social activities could increase their feeling of meaningfulness in everyday life and strengthen their SOC and identity as whole persons.

### Identity and existential issues

The informants in this study were concerned with existential issues such as *hope, life and death*, and *identity*. These siblings’ concerns about existential questions have also been described in previous studies (Long et al., 2015; Nolbris et al., 2007; Tasker & Stonebridge, 2016). Adolescent siblings of children with cancer might have various earlier experiences; however, it could be the first time that they are concerned with such questions. In adolescence, the ability to perform abstract thinking increases (Piaget et al., 1974), and the ability to relate to these questions will depend on the stage of this development. Age-appropriate social and professional support can help siblings reflect on existential questions. Informants described themselves as more empathic than earlier, and more grateful that they and others were healthy, which they could no longer take for granted. Such reflections and changes can provide personal growth and are closely linked to identity: a crucial resistance resource (Antonovsky, 1979). In addition, being more empathic and grateful, as crucial resistance resources, contributed to better social relationships.

### Strengths and limitations

This study had strengths and limitations. One strength was that, to the authors’ best knowledge, this is the first study that has explored the experiences of siblings of children and adolescents with cancer from a salutogenic health-promoting perspective. Thus, the theory of salutogenesis has been applied in a new setting.

One limitation is that the recruitment resulted in only seven informants. Recruiting adolescents is a challenge, given that adolescence may be a difficult time with major changes. In some cases, the parents reported that the requested siblings who did not sign up had expressed that they “did not bother,” they thought it was “lame,” and they were “tired of cancer.” These statements correspond with the finding that siblings can feel forgotten and jealous because of the focus on the sick child. When the treatment is finished, it may be important for the sibling to move on and put the experience behind them. In addition, siblings emphasized in the interviews that they preferred not to be identified with the cancer disease. These could be possible reasons why so few young people signed up. Nevertheless, those who participated were motivated to share their experiences, and the interviews resulted in rich data with both similarities and differences. Another limitation is that all the informants were girls, and the findings might have been different if the sample had covered both genders. However, McDonald et al. (2015) searched for factors that could predict mental difficulties and unmet needs of adolescents and young adults who had a sibling with cancer, and found no significant differences related to the gender or age of the healthy siblings. Nolbris and Ahlström (2014) also found no differences in experiences related to siblings’ gender and age in such contexts. Nonetheless, it can be assumed that affected boys might use other coping strategies than girls, and that siblings who are younger than the sick children handle the stressors in a different way than the older ones. Accordingly, we need more research about male and younger siblings’ access to and use of coping resources.

## Conclusions

The aim of this study was to explore siblings’ experiences with cancer in a young family member in a salutogenic health promoting perspective. The informants described comprehensive changes in their daily lives, feelings, and relationships. Feelings of loneliness and a need for understanding were of particular prominence. The informants stated that social support was a crucial resource to achieve understanding, faith and hope, and identity, which were important coping resources for them. Thus, these siblings’ experiences were compatible with the four crucial spheres: feelings, relationships, activity, and existential questions, all of which—according to the theory of salutogenesis—are keys to developing and strengthening meaning and SOC. This means that resolving tensions by using these resistance resources might promote siblings’ SOC and coping, and maintain or improve their health. The siblings’ ability to find and make use of resistance resources depended on their access to such resources, and on their age and developmental stage. Healthcare professionals should pay close attention to such siblings and their needs, and offer them individually facilitated social support.

This study has demonstrated the significance of salutogenesis in a new setting, and the findings could be of relevance to teachers and different health professionals consulting with the siblings of children with cancer. Further research should be conducted to pinpoint concrete health-promoting measures beneficial to this group.
